# Supplementary material for: Spider-silk inspired polymeric networks by harnessing the mechanical potential of β-sheets through network guided assembly
Source: Nat Commun. 2020 Apr 2;11:1630. doi: 10.1038/s41467-020-15312-x (PMC7118121; doi:10.1038/s41467-020-15312-x)
Supplement: Supplementary file 1 — Supplementary Information [file 41467_2020_15312_MOESM1_ESM.pdf]

Supplementary Information

Spider-silk inspired polymeric networks by harnessing the mechanical potential of β-sheets through network guided assembly

Nicholas Jun-An Chan, et al.

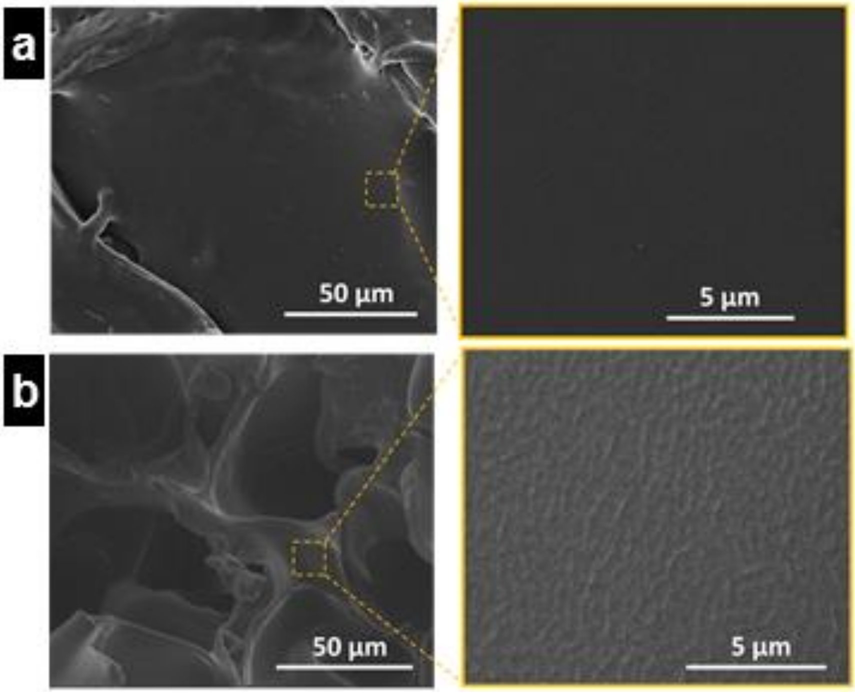

Supplementary Figure 1. SEM cross-sectional images of initial networks at various magnifications (from left to right, scale bars are 50 µm, 5 µm with increasing magnification) with different morphologies showing **(a)** hydrogel and **(b)** cryogels.

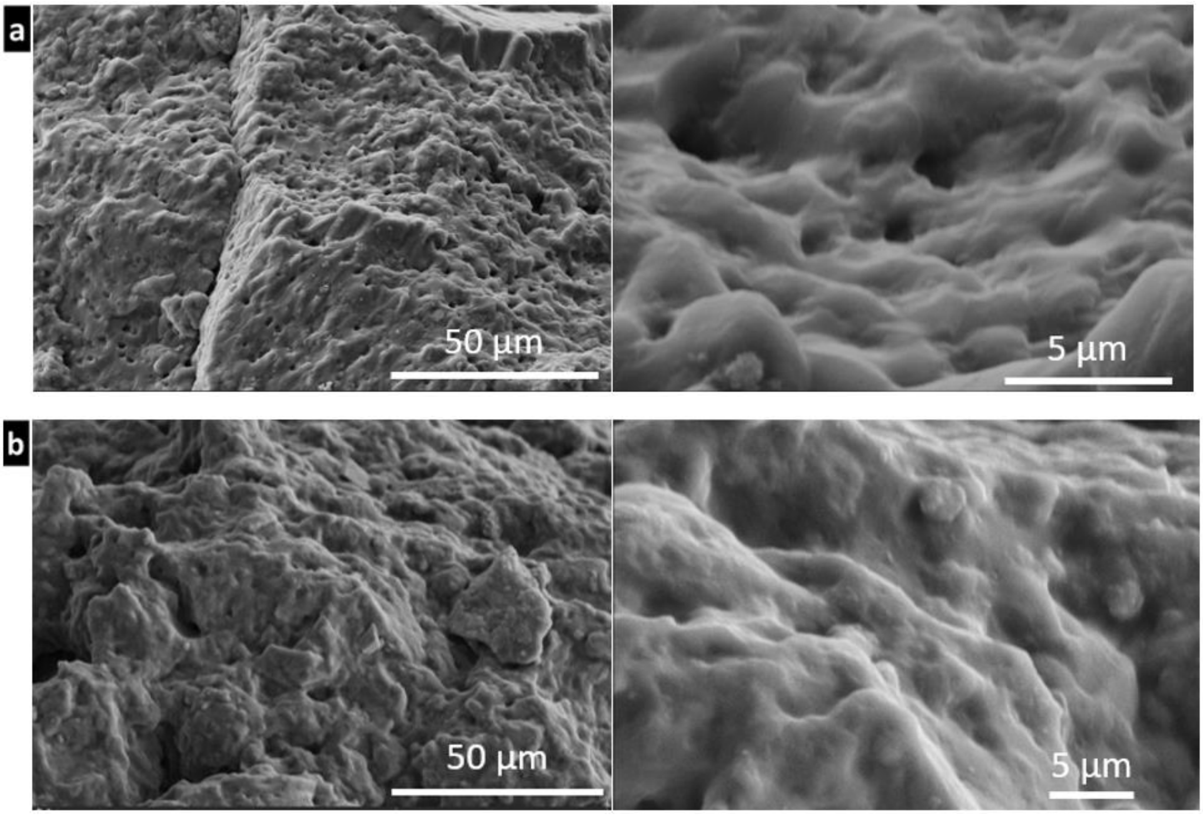

Supplementary Figure 2. SEM cross-sectional images of β-sheets incorporated hydrogel networks at various magnifications (from left to right, scale bars are 50 µm, 5 µm with increasing magnification): **(a)** 50:50 valine to glycine monomer at 0.8M (~120 mg/mL of val NCA); **(b)** glycine 100% 0.8M.

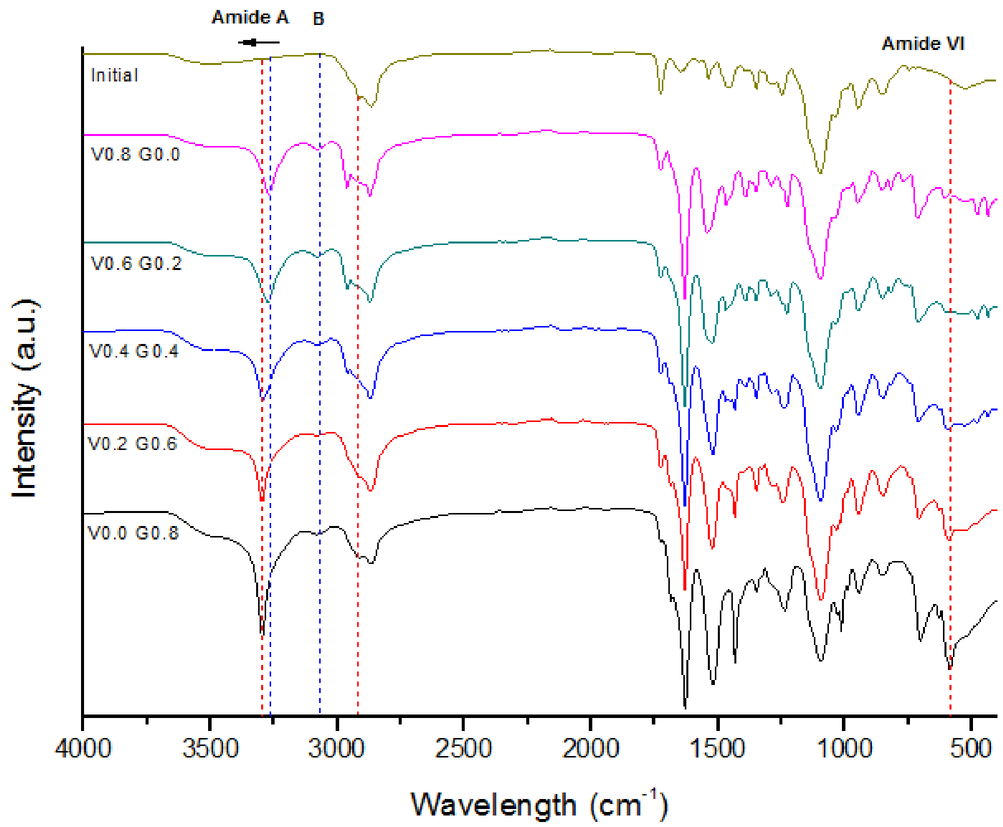

Supplementary Figure 3. ATR-FTIR Spectra showing characterizations of initial network and β-sheet (400 – 4000 cm^{-1}) incorporated final networks obtained using different glycine concentrations at a constant monomer molar concentration (0.8M)

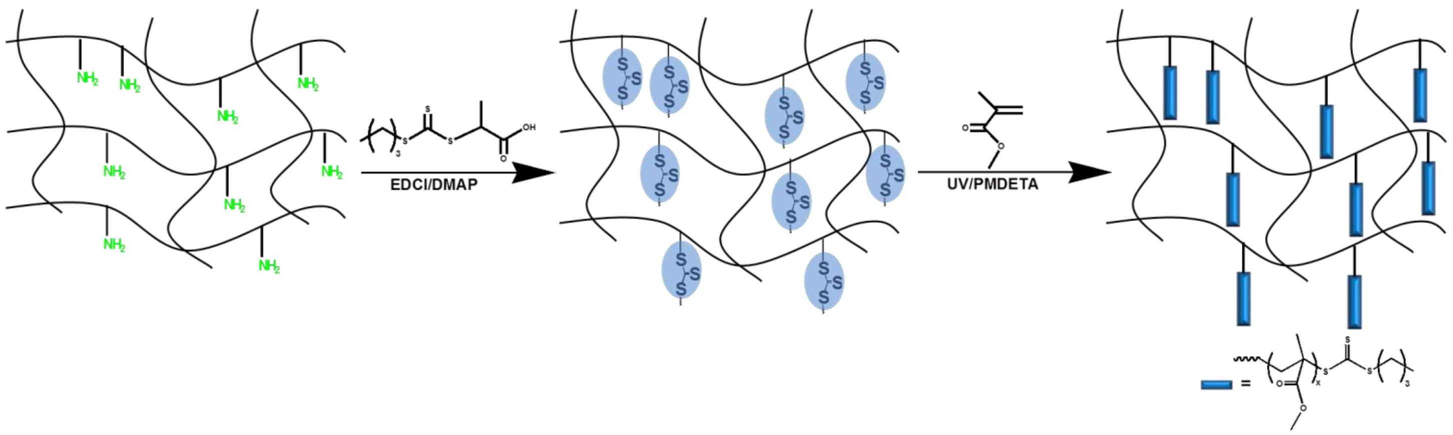

Supplementary Figure 4. Preparation of PMMA-grafted networks (hydrogels & cryogels) through functionalization of initial network with trithiocarbonate iniferters (TTC); **ii)** insertion of PMMA chains via UV-initiated RAFT polymerization based on the attached TTC iniferters, in the presence of MMA monomers at three different concentrations (30, 120, and 360 mg/mL).

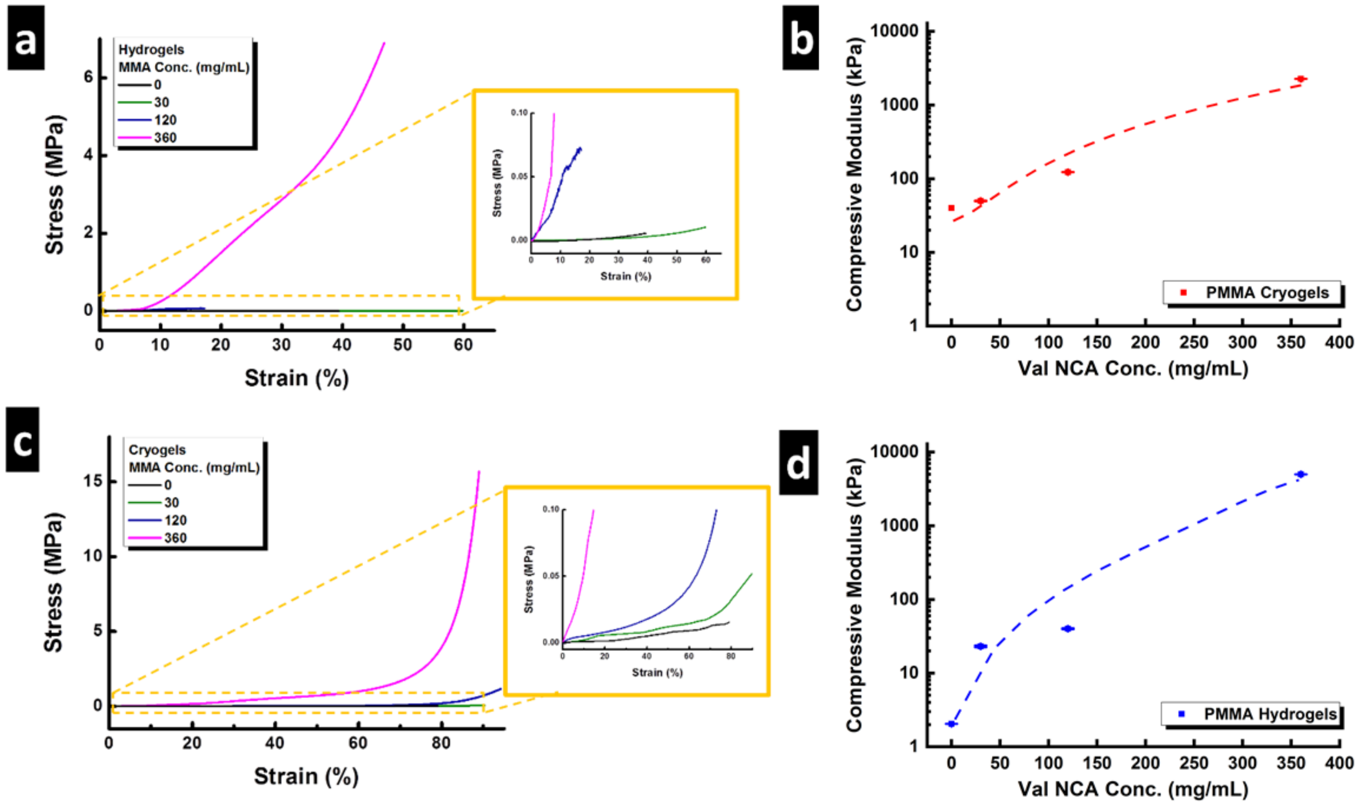

Supplementary Figure 5 (a) & **(c)** Compression strain-stress curves of PMMA grafted networks, hydrogels and cryogels respectively, prepared with different MMA concentrations (30, 120, and 360 mg/mL); **(b)** & **(d)** compression moduli (stiffness) of PMMA grafted networks vs. those of β-sheets incorporated counterparts, hydrogels and cryogels respectively, as a function of corresponding monomer concentrations. Error bars determined by experimental error.

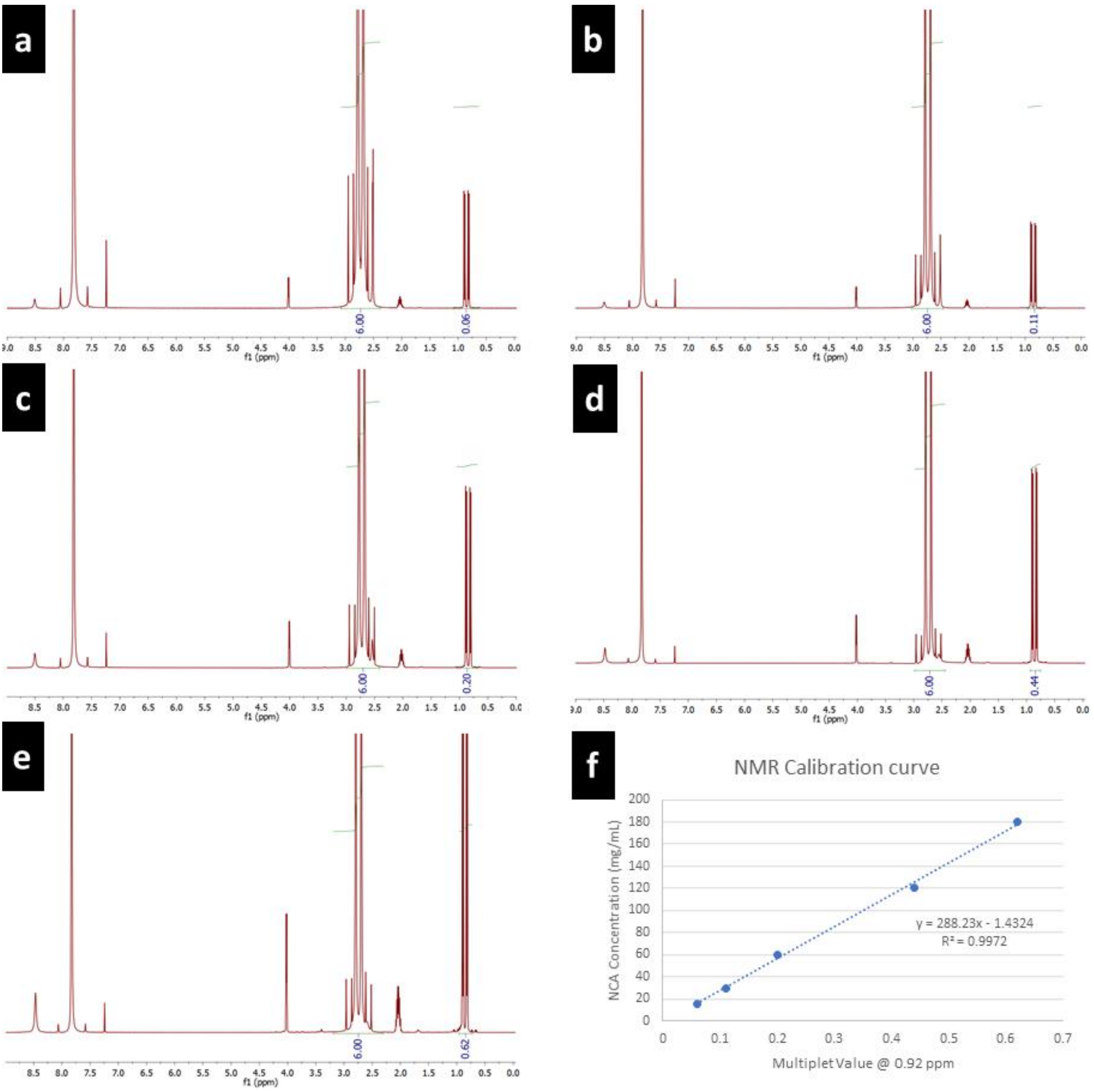

Supplementary Figure 6: H^1 NMR spectra for (a) 15, (b) 30, (c) 60, (d) 120 and (e) 180 mg/mL of Val NCA in DMF with integral of the Val NCA doublet at 0.92 ppm analysed using the two singlets at 2.88 ppm and 2.96 ppm of the two methyl groups from DMF being used to normalize these integrals. (f) Val NCA concentration plotted against value of the integral at 0.92 ppm.

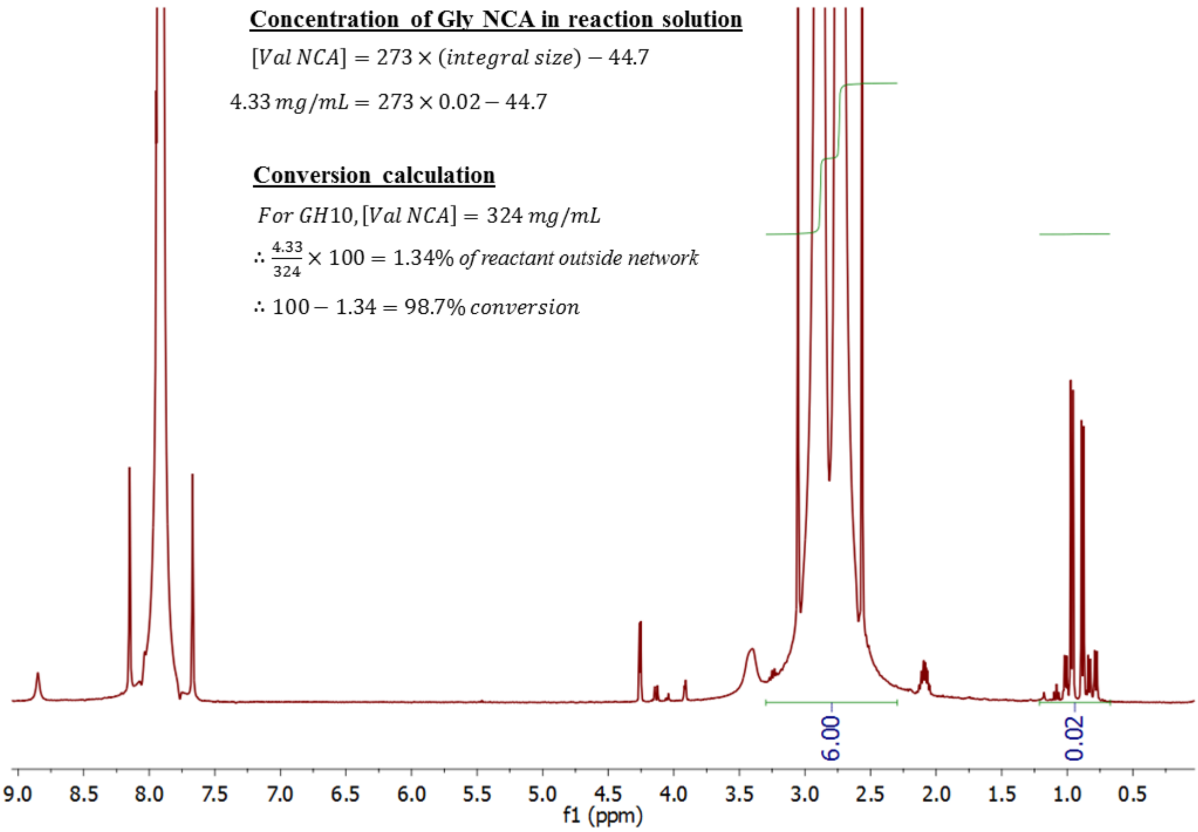

Supplementary Figure 7: ^{1}H NMR spectrum of GH10 reaction solution and calculation of conversion of Val NCA in GH10

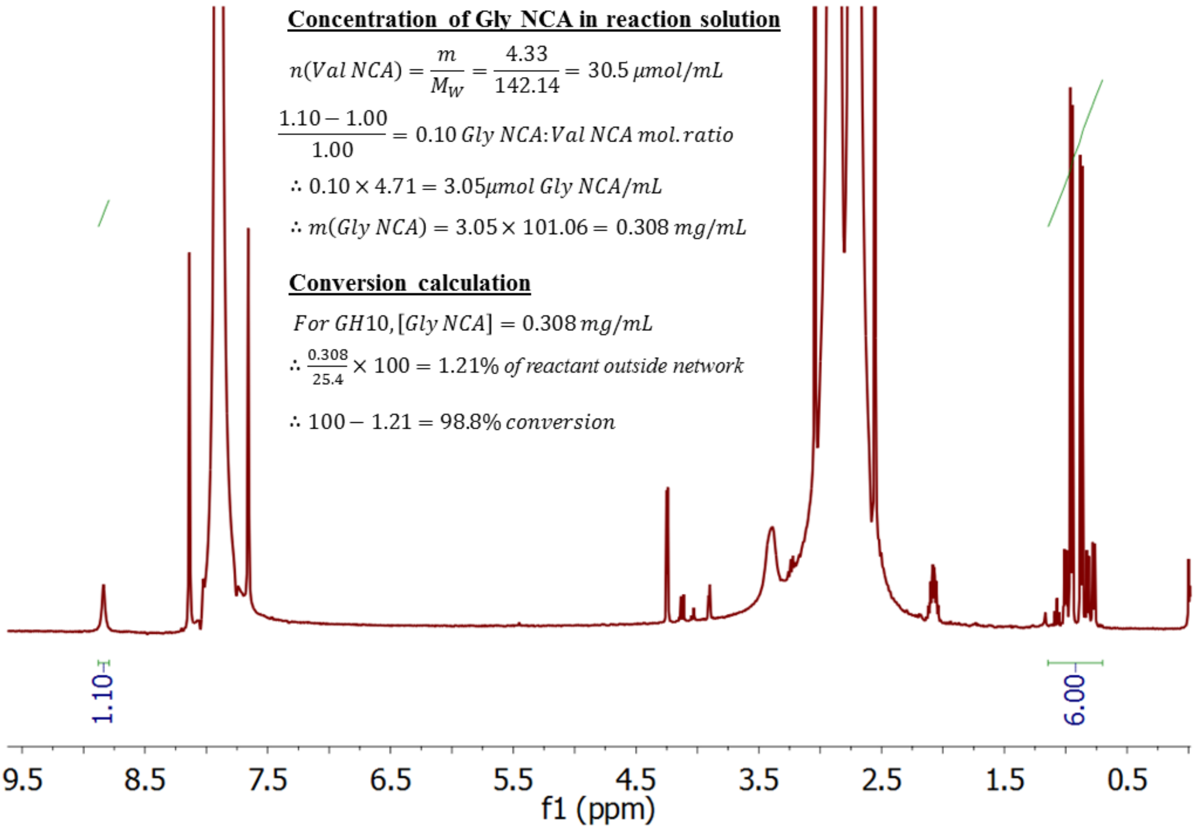

Supplementary Figure 8: ^{1}H NMR spectrum of GH10 reaction solution and calculation of conversion of Gly NCA in GH10
